# A novel method to design and evaluate artificial neural network for thin film thickness measurement traceable to the length standard

**DOI:** 10.1038/s41598-022-06247-y

**Published:** 2022-02-09

**Authors:** Joonyoung Lee, Jonghan Jin

**Affiliations:** 1 Department of Science of Measurement, Korea University of Science and Technology (UST), 217, Gajeong-ro, Yuseong-gu, Daejeon, 34113 Republic of Korea; 2 Division of Physical Metrology, Korea Research Institute of Standards and Science (KRISS), 267, Gajeong-ro, Yuseong-gu, Daejeon, 34113 Republic of Korea

**Keywords:** Optical metrology, Optical sensors

## Abstract

The artificial neural networks (ANNs) have been often used for thin-film thickness measurement, whose performance evaluations were only conducted at the level of simple comparisons with the existing analysis methods. However, it is not an easy and simple way to verify the reliability of an ANN based on international length standards. In this article, we propose for the first time a method by which to design and evaluate an ANN for determining the thickness of the thin film with international standards. The original achievements of this work are to choose parameters of the ANN reasonably and to evaluate the training instead of a simple comparison with conventional methods. To do this, ANNs were built in 12 different cases, and then trained using theoretical spectra. The experimental spectra of the certified reference materials (CRMs) used here served as the validation data of each trained ANN, with the output then compared with a certified value. When both values agree with each other within an expanded uncertainty of the CRMs, the ANN is considered to be reliable. We expect that the proposed method can be useful for evaluating the reliability of ANN in the future.

## Introduction

An artificial neural network (ANN) is a well-known machine learning algorithm used to execute unspecified operations to achieve a certain goal without the operator’s intervention. The ANN is a method that comprehensively utilizes a variety of information by imitating the synapse structure of neurons making up the nervous system^1^. Therefore, the ANN can provide optimized solutions for any type of data. With such advantages, ANNs have been widely used in numerous fields, such as gaming, medicine, image processing, and voice recognition^2^. One of the main characteristics of an ANN is that it can extract meaningful data from complex information that contains noise. Moreover, unlike traditional algorithms that require initial values and multiple iterations, an ANN algorithm provides output in a very short time when trained once in advance.

Metrology throughout semiconductor and display manufacturing processes involves significant amounts of complex data^3–14^. A typical example is to determine the thickness of a thin film using the spectral reflectance spectrum. The reflectance spectrum includes not only information related to the thickness of the thin film but also various effects such as the bandwidth and distribution of the light source, the optical properties of the optical components in use, the uniformity of the material, the linearity of the detectors, environmental fluctuations and external noise. It is practically impossible to extract information of only the thin film thickness from the reflectance spectrum obtained experimentally, which increases measurement uncertainty. In this respect, given the advantages of an ANN, several studies have been reported to measure the thickness of transparent thin films or multiple thin films with a broad spectral light source and X-rays^15–19^. However, in all of these earlier works, the performance evaluation was limited to the level of a simple comparison with the results of existing analysis methods or other measurement techniques. As a result, it was concluded that an ANN works well if the difference between the results from the comparisons is relatively small. Even if the difference is small enough, it doesn’t mean that the ANN algorithm works accurately because both analysis methods can be incorrect. Therefore, because of insufficient verification on the effectiveness of the ANN algorithms, the results obtained with an ANN algorithm should only be used as initial values in a model-based analysis to reduce the difference with less iteration^15,18^. Despite of the practical difficulty regarding analysis reliability, no methods beyond the simple comparison have been proposed and adopted for verifying the ANN algorithms. Besides thickness measurement applications, several studies of ANN algorithms have been also reported in real-life applications related to weather forecasting, yield improvement, and so on^20–24^. Similarly, the performance of the models used in these studies were evaluated in terms of various types of errors like root mean square error between model output and label. Unfortunately, label values based on actual measurements were also basically lack of measurement reliability. The original idea of this work is to exploit the traceability chain of the length standard of SI unit for evaluating the ANN algorithm for the first time. The reasonable determination of the ANN parameters and the reliable evaluation of the ANN algorithm can be achieved based on this concept, not using simple comparison with conventional methods.

In this article, an ANN algorithm for thin film thickness measurements was designed and verified using four certified reference materials (CRMs). Certification was done using a standard instrument based on spectral ellipsometry at the Korea Research Institute of Standards and Science. The ultimate goals of this study are (1) reasonable selection of the parameters of ANN algorithms and (2) a performance evaluation based on an international standard instead of a simple comparison with current analysis methods or measurement techniques. To accomplish this, ANNs for the thin film thickness analysis were built in 12 conditions with different combinations of the number of hidden layers (L = 1, 2, 3) and the number of nodes in each layer (N = 50, 100, 150, 200). These 12 ANN algorithms were trained in a thickness range of 1–110 nm. The training spectra were created by a numerical simulation based on a multiple interference within the thin film. To evaluate the reliability of each ANN algorithm, the measured spectra of CRMs with nominal thicknesses of 10 nm (CRM-10), 30 nm (CRM-30), 50 nm (CRM-50), and 100 nm (CRM-100) were used as the validation data sets. When a trained ANN algorithm provides outputs that are in good agreement with the certified values from the CRMs, it is considered to function properly. To the best of our knowledge, this study is the first to evaluate ANN algorithms intended to measure thin film thickness value based on the traceability chain of a length standard. It is very difficult to conclude that the simple comparison methods in previous works provide measurement reliability applicable to metrology.

## Methods

**Figure 1:**
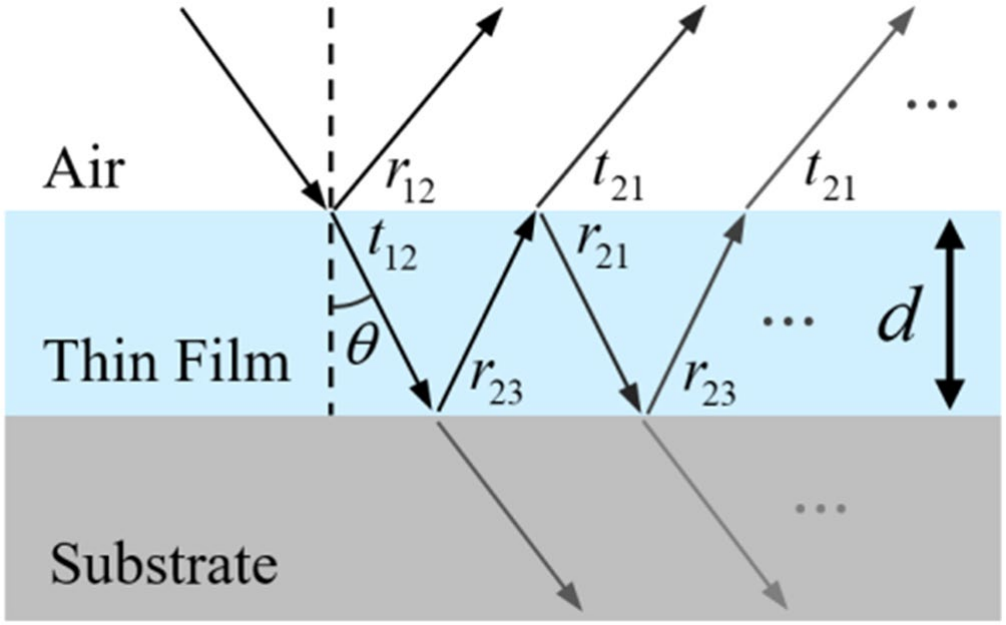
Schematic diagram of multiple reflections and transmissions taking place within the thin film layer of a specimen (*r*_12_: Fresnel reflection coefficient of light incident from air to the film, *t*_12_: Fresnel transmission coefficient of light incident from air to the film, *r*_23_: Fresnel reflection coefficient of light incident from the film to the substrate, *r*_21_: Fresnel reflection coefficient of light incident from the film to air, *t*_21_: Fresnel transmission coefficient of light incident from the film to air, *d*: Film thickness).

For thin film thickness measurements, the spectral reflectometer has been widely employed due to high measurement speed and simple configuration^3–7,12,14^. The lights reflected from the top and bottom surfaces interfere with each other, as shown in Fig. 1. These lights usually have a wide spectral bandwidth covering the whole visible range and can be emitted from gas lamps such as a tungsten-halogen lamp. The reflected lights can be detected by a visible spectrometer in a form of a spectrum, the mathematical model of which can be expressed by Eq. (1). The reflectance spectrum depends on the thickness of the thin film (*d*) as well as the Fresnel reflection coefficients at the interfaces (*r*_12_ and *r*_23_), the complex refractive index of medium ($\tilde{N }(k)$), the wavenumber (*k*) and the refracted angle (*θ*) according to the Fresnel equation^14^.

1 $$R(d;k) = \frac{{{r_{12}} + {r_{23}} \cdot {e^{ - j \cdot 2 \cdot k \cdot d \cdot \tilde N(k) \cdot \cos \theta }}}}{{1 + {r_{12}} \cdot {r_{23}} \cdot {e^{ - j \cdot 2 \cdot k \cdot d \cdot \tilde N(k) \cdot \cos \theta }}}}$$

When the reflectance spectrum at an unknown thickness is obtained experimentally, the thickness cannot be easily determined by analyzing the spectrum itself. In such a case, each measured spectrum is compared individually with many spectra generated by Eq. (1). The reflectance spectra are generated with a certain thickness step in the measurable thickness range. In the absence of an estimated thickness of the specimen, a large number of reflectance spectra are required to find the optimal thickness in a wide arbitrary thickness range. Moreover, to improve the thickness measurement resolution during this comparison, the number of spectra should increase by adopting a smaller thickness step. Therefore, for a precise analysis of an arbitrary thickness, this model-based algorithm requires a considerable amount of time during the comparison process. After this comparison, the thickness used to generate the spectrum with the least square error relative to the measured spectrum is chosen as the solution of the thickness of the thin film. When the thin film thickness is outside the range of comparison, a solution may not be found, or an incorrect thickness value may be obtained. On the other hand, the learned ANN algorithm instantly gives the analyzed thickness value of a given reflectance spectrum with no need of multiple iterations and an initial value. Therefore, the ANN algorithm can be useful for real-time applications.

**Figure 2:**
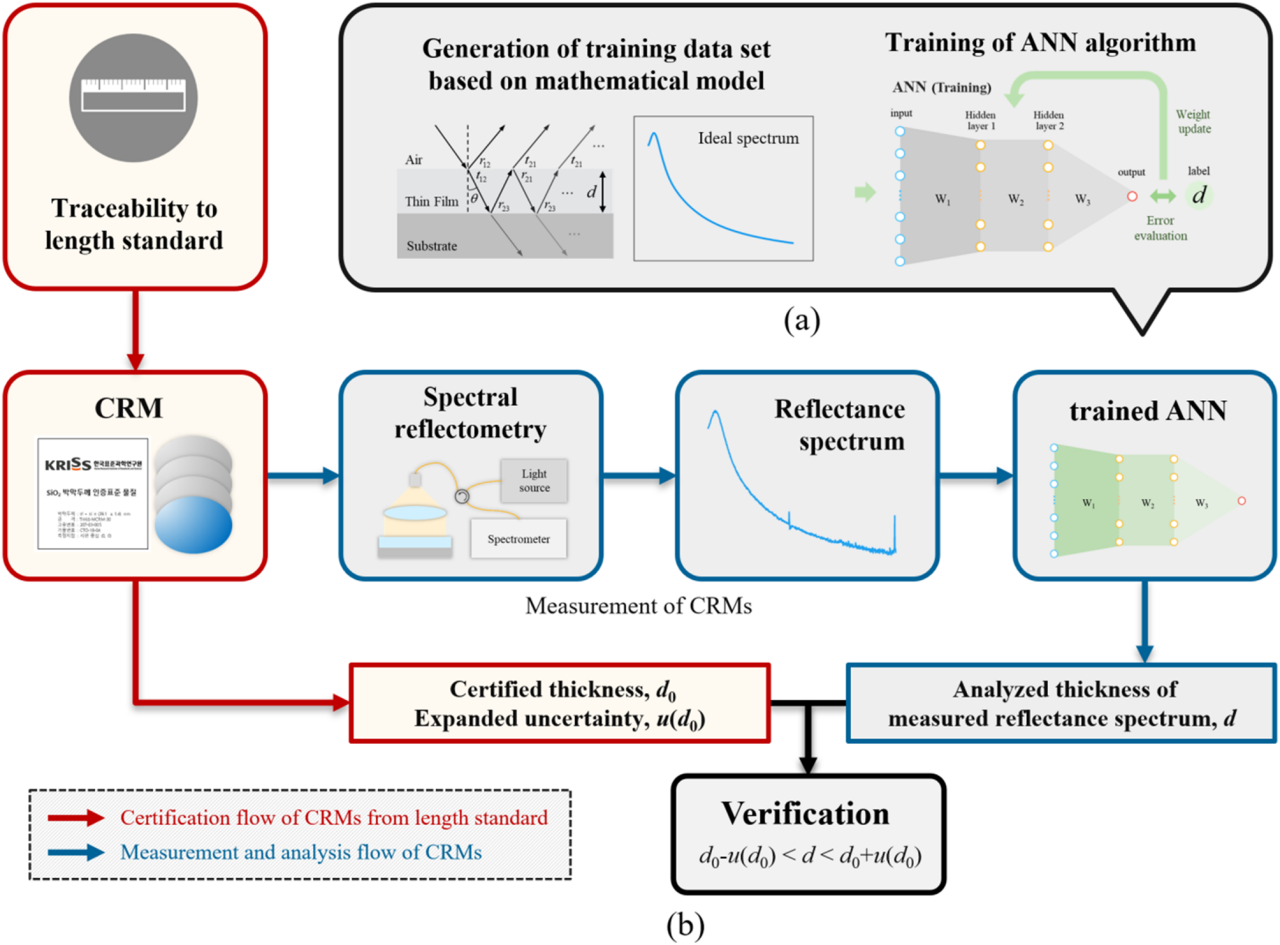
Schematics of the proposed method: (**a**) conventional training process using numerical simulations. The ideal reflectance spectrum for the thin film thickness *d* is used as the input, and the *d* is used as the correct label. (**b**) Flowchart of the proposed method for design and verification of ANN algorithm for measuring thin film thicknesses.

Figure 2 shows the schematics of the proposed method for the design and verification of an ANN algorithm. In Fig. 2a, the conventional method of training for an ANN algorithm is shown, matching those in previous works^15–19^. A multilayer perceptron (MLP) type ANN algorithm was constructed and trained using Python, similarly to the previous work^15,17,19^. In the wavelength range of the spectrometer to be used for the CRM measurement, a wavelength range in which the intensity of the measured light is sufficiently greater than noise was selected, and the number of samples for that range was established as the number of input nodes. Therefore, reflectance spectra are received from 881 input nodes and thin film thickness analysis values are output from one output node through a hidden layer. A sigmoid function was applied as the activation function like the previous works^15,19^, and the loss was calculated according to the average of the mean squared error between the outputs and the ideal value as determined in the simulation. After the error estimation is completed, a basic backpropagation algorithm is utilized to update the weights connecting each layer. Batch gradient descent was used with a learning rate of 0.000001. For weight initialization, Xavier initialization was utilized. Because the purpose of this study is not to improve the performance of ANN algorithms, of which basic form is only exploited without any additional techniques among other advanced algorithms. In our work, in the thickness range of 1–110 nm, 110 reflectance spectra were numerically generated with equal steps of 1 nm based on a mathematical model with the Fresnel equation, as expressed by Eq. (1). The wavelength range of the spectrum was 355–657 nm with 881 sampling points. In this case, 70% of the ideally created reflectance spectra were utilized as a training dataset, and the others were a test dataset of the ANN algorithms. Because the proposed method can basically offer strict validation of the ANN algorithms using certified values of the CRMs, it is not necessary to use validation data separately from ideally created reflectance spectra used for training datasets. The design parameters of the ANN algorithm were number of hidden layers (L = 1, 2, 3) and number of nodes in each layer (N = 50, 100, 150, 200), which were selected as simple cases based on the previous works^15,17,19^. Therefore, with the combination of these two parameters, 12 ANN algorithms were developed and then trained.

**Figure 3:**
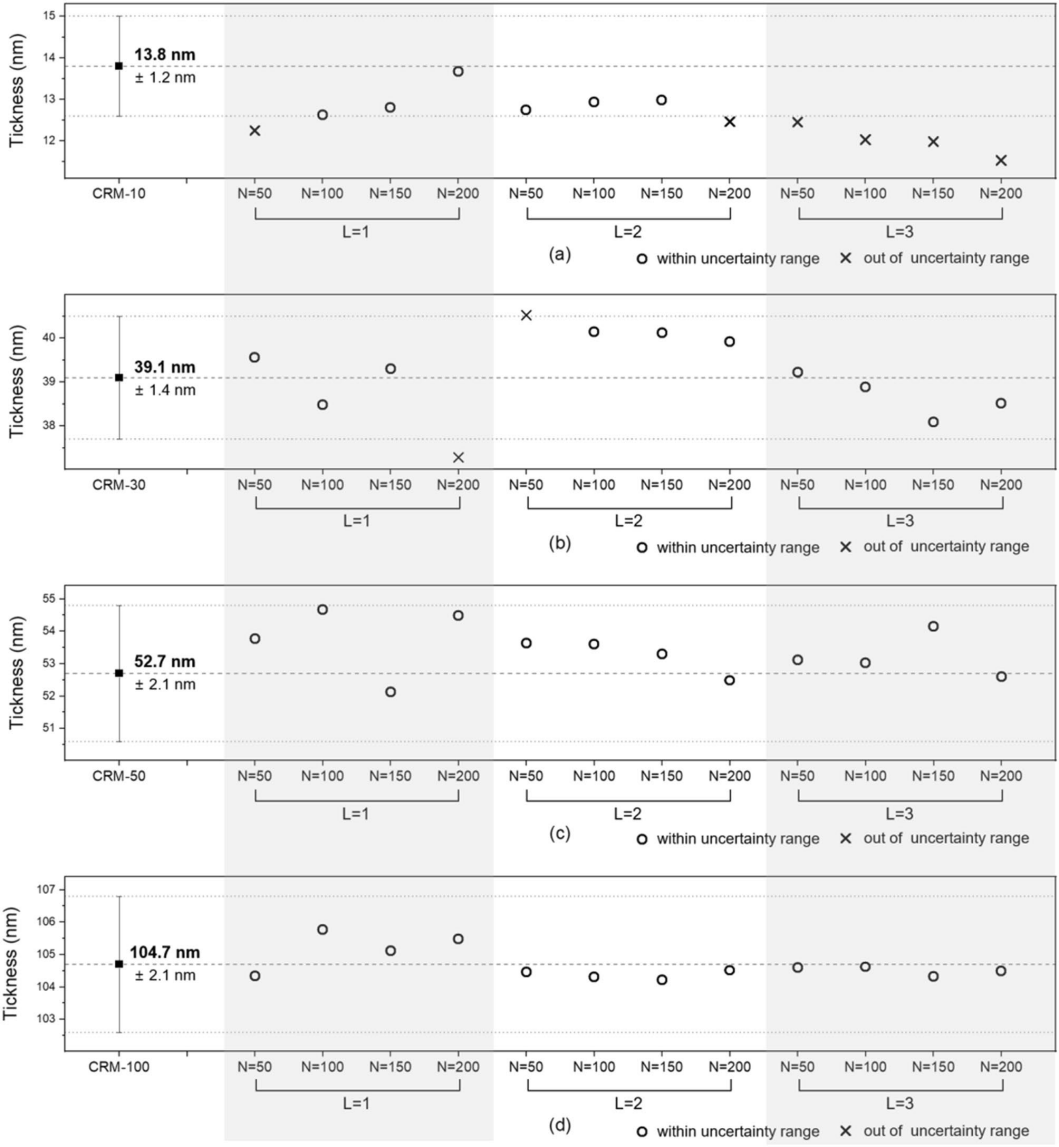
Analyzed thickness values of trained ANN algorithms for (**a**) CRM-10, (**b**) CRM-30, (**c**) CRM-50, (**d**) CRM-100.

Figure 2b shows the procedure used to measure the reflectance spectrum of the CRMs and to verify the trained ANN algorithms. Considering the thickness measurement range, four CRMs of CRM-10, CRM-30, CRM-50, and CRM-100 were exploited to verify the 12 ANN algorithms. The CRMs have two layers consisting of a silicon dioxide (SiO_2_) thin film on a thick silicon substrate. Table 1 shows the certified values and expanded uncertainties of each CRM in use, as provided by the Korea Research Institute of Standards and Science. In our experiments, the reflectance spectra of these CRMs were obtained using a commercial spectrometer with a wavelength range of 190–879 nm with 2048 sampling points. Any other spectrometers detecting the wavelength range of 300–700 nm can be used for determination of thin-film thickness in spectral reflectometry^3,5,7,12,14^. The Deuterium lamp used as a light source emits white light in a wide wavelength range of 112–900 nm. The light was normally incident with regard to the thin film surface with a beam diameter of 2.6 mm in the form of plane wave created using a collimating lens, as shown in the spectral reflectometry part in Fig. 2b. The reflectance spectra obtained experimentally using the four CRMs served as the validation data of the trained ANN algorithms. The outputs of the trained ANN algorithms were plotted for a comparison with certified values from the four CRMs, as shown in Fig. 3. When the outputs agreed with all of these certified values of the CRMs, it was concluded that the trained ANN algorithm works properly.

**Table 1:** Summary of CRMs.

Sample number	CRM number	Materials	Nominal value (nm)	Certified value (nm)	Expanded uncertainty (*k* = 2) (nm)
CRM-10	207-03-006	SiO_2_ thin film on Si wafer	10	13.8	1.2
CRM-30	207-03-005		30	39.1	1.4
CRM-50	207-03-004		50	52.7	2.1
CRM-100	207-03-003		100	104.7	2.1

## Results and discussion

For clarity, Table 2 summarizes the comparison results from Fig. 3 in terms of reliability and effectiveness. The columns shown in diagonal represent the output of each ANN algorithm when in disagreement with the corresponding certified value. In this study, certified values and expanded uncertainties of the CRMs in use were adopted as the quantitative evaluation criteria. The certified values were determined through a rigorous measurement process, which fully satisfies the traceability system of the international standard. During the measurements, even if this task follows a rigorous process, various types of uncertainties can always occur due to unstable environmental conditions. Lots of uncertainty components can affect the measurement result all together, which is expressed as an expanded uncertainty (approximately 95% confidence level of the certified value, coverage factor k = 2). Based on this quantitative evaluation criteria, only four cases consisting of 150 nodes and 200 nodes with one hidden layer (N = 150, 200 with L = 1) and two hidden layers (N = 150, 200 with L = 2) were chosen as trustworthy candidates. For a quantitative comparison of cases, the offset between the outputs and the certified values of the CRMs were calculated and then averaged. The averaged offsets (*δ*) in cases were 1.2 nm for 100 nodes with one hidden layer (N = 100 with L = 1) and 0.54 nm for 150 nodes with one hidden layer (N = 150 with L = 1) and 0.81 nm for 100 nodes with two hidden layers (N = 100 with L = 2) and 0.74 nm for 150 nodes with two hidden layers (N = 150 with L = 2). Thus, we finally selected the ANN algorithm with the lowest value of the averaged offset, which in this case was 150 nodes with one hidden layer (N = 150 with L = 1). Unexpectedly, the ANN algorithms with more hidden layers, i.e., L = 3, and more nodes, i.e., N = 200, did not always result in better reliability. In the authors’ view, the results showed that the ANN algorithms with more nodes and hidden layers were over-trained by only the training data set of ideally generated reflectance spectra, which may result in overfitting. Hence, the proposed method can be beneficial for evaluating accuracy of the ANN algorithms with the help of a traceability chain of a length standard.

**Table 2:**
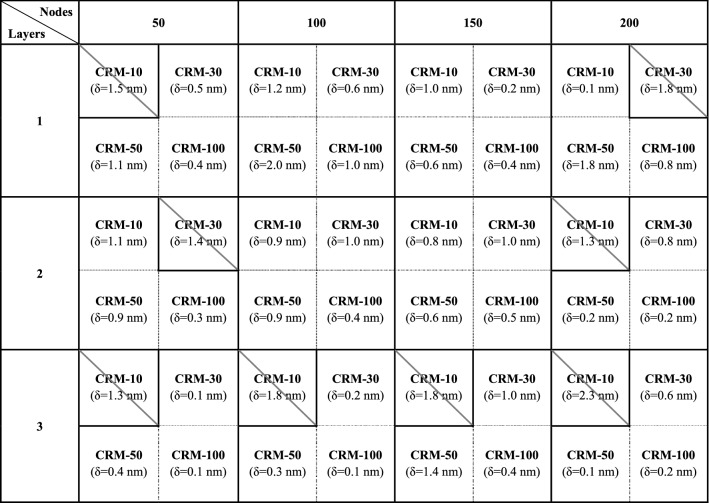
Summary of verification of the ANN algorithms.

Diagonal down bordered column represents that the analyzed value not agreed with the corresponding certified value.

More importantly, in some previous works, when the difference between the ANN algorithm and the model-based algorithm was found to be relatively large, the output of the ANN algorithm was used as the initial value of the model-based algorithm to reduce the difference^15,18^. This allows the iteration steps of the model-based algorithm to be reduced. In such case, the output of the model-based algorithm was used as a reference value or as a true value regardless of its reliability. The smallest deviation between two outputs cannot always mean that the ANN algorithm is designed well and works properly.

## Summary

In this article, a novel method to design and evaluate an ANN algorithm used to determine the thickness of thin films was proposed and demonstrated. As a reference value, a CRM certified value directly traceable to a length standard was utilized. Twelve ANN algorithms with different conditions (L = 1, 2, 3 and N = 50, 100, 150, 200) were developed in-house and then trained using 110 numerically created reflectance spectra in a thickness range of 1–110 nm. With reflectance spectra of the 4 different CRMs obtained by experiments, the thickness values were determined by 12 well-trained ANN algorithms and then compared with the corresponding certified values of the CRMs. As a result, based on a traceability chain to the length standard, each ANN algorithm was evaluated. Finally, in this work, an ANN algorithm with 150 nodes with one hidden layer was chosen as the best case with an average offset of 0.54 nm, as derived from the differences between the outputs and the certified values of the CRMs. The practical applications of this study can be limited to only cases providing certified values for the present. It is expected that the proposed approach will be beneficial for those involved in developing and verifying machine-learning algorithms for rigorous metrology. In the future, for completeness of this study in a metrological view, uncertainty evaluations need to be performed according to Guide to the Expression of Uncertainty in Measurement.

## Data Availability

The datasets generated during the current study are available from the corresponding author on reasonable request.
